# Congenital atrophic dermatofibrosarcoma protuberans detected by COL1A1-PDGFB rearrangement

**DOI:** 10.1186/s13000-016-0474-6

**Published:** 2016-03-01

**Authors:** Maki Makino, Shunsuke Sasaoka, Gen Nakanishi, Eiichi Makino, Wataru Fujimoto

**Affiliations:** Department of Dermatology, Kawasaki Medical School, 577 Matsushima, Kurashiki-shi, Okayama-ken, 701-0192 Japan; Department of Dermatology, Shiga University of Medical Science, Seta Tsukinowa-cho, Otsu-shi, Shiga-ken 520-2192 Japan; Department of Dermatology, Kawasaki Hospital, Kawasaki Medical School, 2-1-80 Nakasange, Kita-ku, Okayama-shi, Okayama-ken, Okayama 700-8505 Japan

## Abstract

**Background:**

Atrophic variant of dermatofibrosarcoma protuberans (DFSP) is a distinct form of DFSP.

**Case presentation:**

Here, we report the case of a 19-year-old woman with a small congenital atrophic plaque on the right precordium. The lesion remained atrophic for more than 10 years. Several years earlier, a portion of the plaque became tuberous and enlarged. Physical examination revealed a 25 × 30 mm erythematous atrophic plaque surrounded by three hard, smooth, and orange-colored nodules of varying sizes on the right precordium, along with visible subcutaneous adipose tissue and cutaneous veins. Biopsy of the nodule and atrophic plaque revealed dense proliferation of spindle-shaped tumor cells from the dermis to the subcutaneous adipose tissue, and positive immunostaining for CD34 and vimentin in addition to negative staining for factor XIIIa and α-smooth muscle actin. Reverse transcription polymerase chain reaction (RT-PCR) of the tumor tissue revealed the presence of a *COL1A1-PDGFB* fusion gene. Thus, congenital atrophic dermatofibrosarcoma protuberans was diagnosed. No metastasis to the lungs or regional lymph nodes was found on magnetic resonance imaging. Wide local excision and split-thickness skin grafting was performed and neither recurrence nor metastasis has been observed for 5 years and 8 months since the surgery.

**Conclusion:**

This case indicates that a congenital atrophic lesion could represent a quiescent phase of DFSP. Awareness of this rare condition can aid with early diagnosis and thereby improve the prognosis of DFSP.

## Background

Dermatofibrosarcoma protuberans (DFSP) is a locally aggressive mesenchymal neoplasm of the skin characterized by high rates of local recurrence after surgical excision but a low risk of metastasis to the lymph nodes and other organs [1]. DFSP is cytogenetically featured by t(17;22)(q22;q13) translocation, resulting in the fusion of the collagen type I α1-chain (*COL1A1*) gene with the platelet-derived growth factor β-chain (*PDGFB*) gene [1–5]. The *COL1A1-PDGFB* fusion gene is detected in >90 % of cases of DFSP [1, 4] and represents a very useful tool for the differential diagnosis of DFSP with other benign/malignant neoplasms.

The DFSP lesion is usually present as a tumor that protrudes above the surface of the skin. Atrophic DFSP, a recently identified rare variant of DFSP, appears as a depressed plaque [6–10]. In this variant, the fusion gene has been detected in three reported cases [2, 5, 11]. This rare variant is considered an early stage of DFSP [8]. Here we describe the case of a 19-year-old woman with congenital atrophic DFSP on the right precordium. The lesion was recognized at birth and remained atrophic for more than 10 years before developing into a tumor. The presence of a *COL1A1(exon25)-PDGFB(exon2)* fusion gene detected by polymerase chain reaction (PCR) confirmed the diagnosis of congenital atrophic DFSP. Recognition of this rare atrophic variant of DFSP can lead to early diagnosis and to improve the disease prognosis.

The goal of this report is to highlight the potential clinical variability in the presentation of congenital DFSP and the importance of considering this diagnosis in pediatric patients with atypical cutaneous or subcutaneous tumors.

## Case presentation

**Fig. 1:**
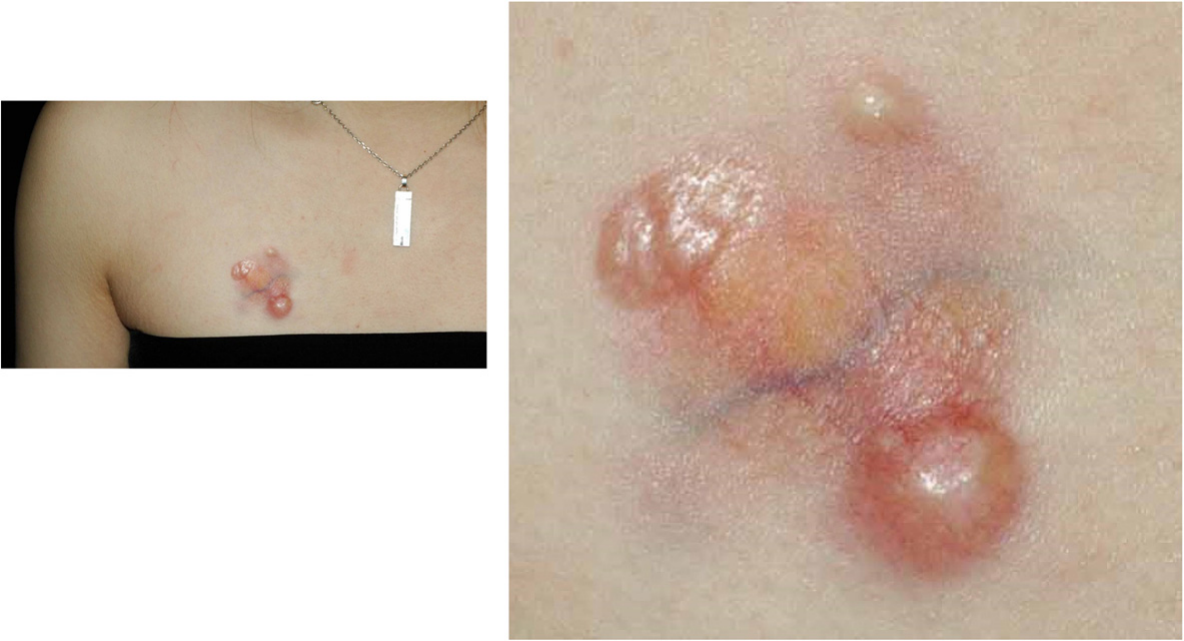
A 25 x 30 mm erythematous atrophic plaque was seen on the right precordium, along with visible subcutaneous adipose tissue and cutaneous veins

A 19-year-old woman presented with an atrophic plaque and small nodules on the right precordium. The atrophic plaque was present at birth and remained quiescent for more than 10 years. Several years prior, a portion of the plaque became tuberous and enlarged, and the patient presented to our institution. She was otherwise healthy and had no systemic symptoms and no remarkable family history. On initial examination, a 25 × 30 mm erythematous atrophic plaque was seen on the right precordium, along with visible subcutaneous adipose tissue and cutaneous veins (Fig. 1). Three hard, smooth, and orange-colored nodules of varying sizes were found around the plaque. The nodules were mobile and not affixed to the deeper structures.

**Fig. 2:**
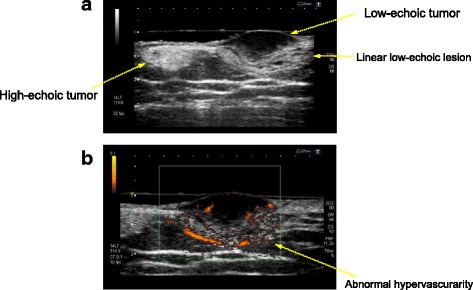
Ultrasonography revealed both hyper- and hypo- echoic tumors from the superficial dermis to the subcutaneous tissue (**a**). Both lesions were ill-defined and hypo-echoic tumors were suspected to be malignant because of their abnormal hypervascularity (**b**)

**Fig. 3:**
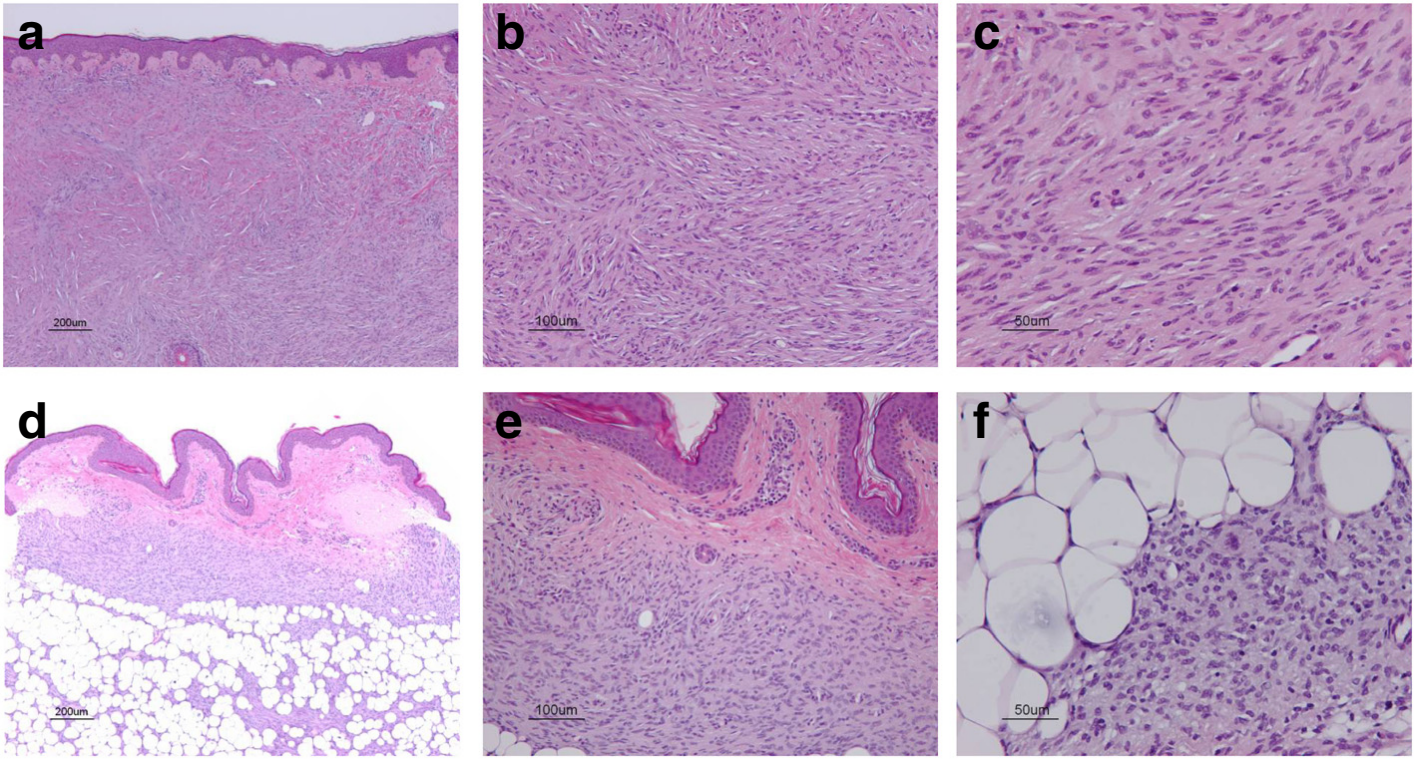
Biopsy of the nodule revealed dense proliferation of spindle-shaped tumor cells from the dermis to the subcutaneous adipose tissue, and in some areas, densely packed spindle cells were arranged in a storiform pattern (**a**-**c**). Biopsy of the atrophic plaque revealed that the thickness of the dermis is reduced, and a spindle cell proliferation replaces the dermis extending into the subcutaneous tissue (**d**-**f**)

**Fig. 4:**
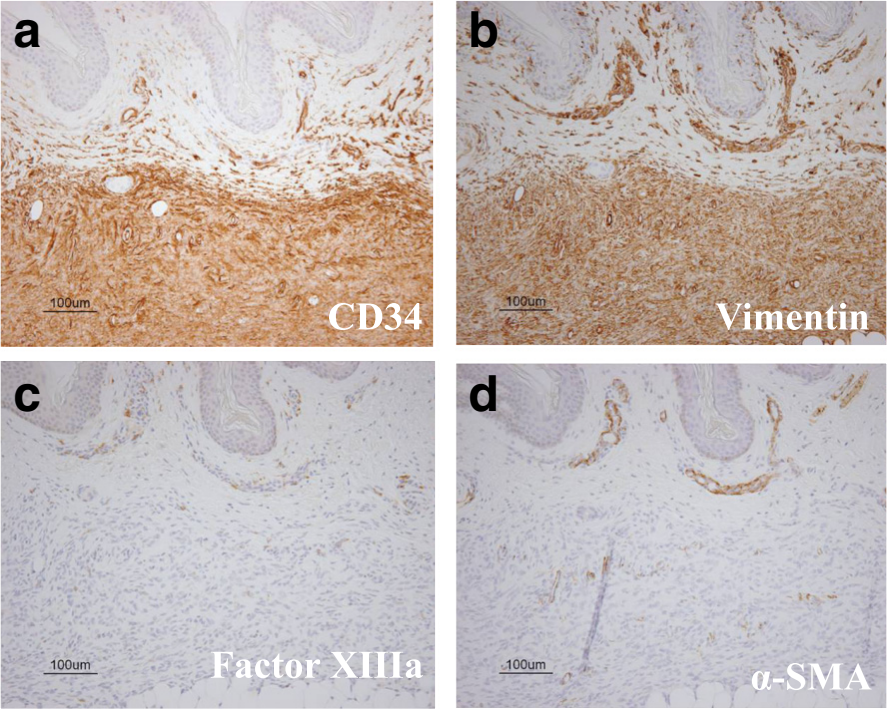
Tomor cells in elevated plaque revealed positive immunostaining for CD34 (**a**) and vimentin (**b**), and negative staining for factor XIIIa (**c**) and α-SMA (**d**)

Ultrasonography revealed both hyper- and hypoechoic tumors from the superficial dermis to the subcutaneous tissue (Fig. 2a). Both lesions were ill defined and the hypoechoic tumors were suspected to be malignant because of their abnormal hypervascularity (Fig. 2b). Biopsy of the nodule revealed dense proliferation of spindle-shaped tumor cells from the dermis to the subcutaneous adipose tissue; in some areas, densely packed spindle cells were arranged in a storiform pattern (Fig. 3a-c). Biopsy of the atrophic plaque showed reduced dermal thickness and spindle cell proliferation replaced the dermis extending into the subcutaneous tissue (Fig. 3d-f). Tumor cells in the elevated and atrophic plaques stained positive for CD34 and vimentin and stained negative for factor XIIIa and α-smooth muscle actin (Fig. 4).

**Fig. 5:**
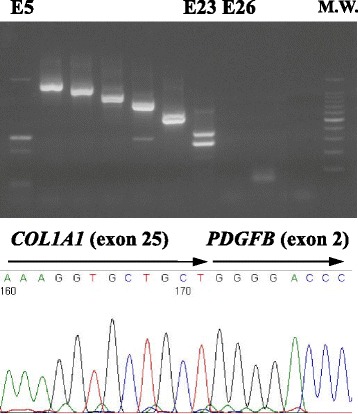
Sequencing of the multiplex RT-PCR amplification product revealed a fusion of exon 25 of *COL1A1* to exon 2 of *PDGFB*

Reverse transcription PCR (RT-PCR) of the tumor tissue revealed gene fusion between exon 25 of *COL1A1* and exon 2 of *PDGFB* (Fig. 5); thus atrophic dermatofibrosarcoma protuberans was diagnosed. Magnetic resonance imaging (MRI) did not reveal any metastasis to the lungs or regional lymph nodes. A wide local excision (25–30 mm) was performed and artificial skin was grafted. After the resection, the stump was confirmed to be negative and the defective area was reconstructed by split-thickness skin grafting. Neither recurrence nor metastasis has been observed for 5 years and 8 months since the surgery.

DFSP, a cutaneous fibrohistiocytic tumor of intermediate malignancy [1], constitutes only 1 % of all soft-tissue sarcomas; nonetheless, it is the most common sarcoma of cutaneous origin [1]. It commonly occurs in individuals 20–50 years of age with an equal sex distribution [1]. DFSP is a slow growing asymptomatic tumor, and some authors believe that many cases diagnosed in adulthood were already present in childhood [1, 8]. In 40–50 % of cases, DFSP preferentially occurs on the trunk, chest, and shoulders [1]. In 30–40 % of cases, the tumor is located in the proximal portion of the limbs; and in 10–15 % of cases, it affects the head and neck [1]. Checketts et al. [12] reported that congenital DFSP was predominantly located on the trunk in 86 % of cases with a male to female ratio of 1:2. The present case was of a woman with an atrophic plaque on the trunk, which matched the features of congenital DFSP described previously.

**Fig. 6:**
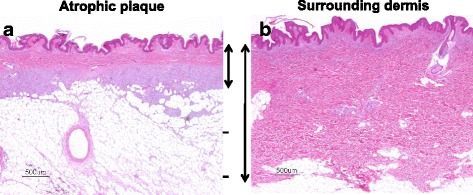
a: atrophic plaque, b: surrounding dermis. Dermal thickness was reduced by 30 %, and tumor cells replace the dermis extending into the subcutaneous tissue

DFSP usually appears as an indurated exophytic plaque that protrudes above the surface of the skin. Some patients with DFSP have atrophic plaques that might be clinically quiescent but have rarely been reported and characterized in detail. The atrophic variant of DFSP was first reported in 1985 by Lambart et al. [6] as dermatofibrosarcoma “non”-protuberans in a patient with the clinical features of morphea and morpheaform basal cell carcinoma. In 1987, Page and Assaad [7] reported five additional cases with DFSP with rare clinical presentations and proposed the term “atrophic DFSP.“ Thereafter, atrophic DFSP has been recognized as a distinct variant of DFSP, and to date, the term “atrophic DFSP“ has been widely used [1, 2, 5, 9, 10]. Atrophic DFSP is characterized by a depressed plaque that persists for a long period of time before developing into a tumor. Histologically, the dermal thickness is reduced by >50 % compared to the surrounding dermis, placing the subcutis close to the epidermis [1]. In the present case, dermal thickness was reduced by 30 % and tumor cells replaced the dermis extending into the subcutaneous tissue (Fig. 6.). Immunohistochemical staining for CD34 and factor XIIIa is of great diagnostic value for DFSP. In a typical case of DFSP, CD34 is positive in 80–100 % of cases, while factor XIIIa is negative in 75 % of cases [1]. In the present case, the staining pattern was typical, CD34 was positive, and factor XIIIa was negative.

Martin et al. [8] analyzed 35 previously published cases of childhood DFSP. They presumed that the so-called “non-protuberant” variant is one of the clinical variants of DFSP that occurs prior to development of the indurated tumor. They classified the early stage of DFSP into four distinct variants: 1) confluent nodular lesions forming a sclerotic plaque, 2) keloid-like homogenous sclerotic plaque, 3) tumor *ab initio*, and 4) atrophic plaque. Hanabusa et al. [13] reported a case of a patient who was initially diagnosed with morphea that subsequently developed into protuberant tumors on the face 20 years later and was finally diagnosed with atrophic DFSP. In the present case, the congenital atrophic plaque remained quiescent for more than 10 years followed by the development of small nodules on the right precordium. This long quiescent phase initiated the thought that the atrophic plaque might represent an early lesion in the course of typical DFSP.

Atrophic DFSP is a soft tissue tumor of intermediate malignancy that has no difference in prognosis compared to typical DFSP [1]. Atrophic DFSP presents as a depressed plaque that can be clinically confused with benign lesions such as morphea, atrophoderma, atrophic scars, anetoderma, and lipoatrophy [1]. Therefore, recognition of the characteristic features of atrophic DFSP is mandatory to avoid misdiagnosis. Medallion-like dermal dendrocyte hamartoma (ML-DDH) is a recently described congenital benign dermal lesion that was first reported by Rodríguez-Jurado et al. [14] in 2004. Clinical, histological, and immunohistochemical findings of ML-DDH are very similar to those of atrophic DFSP. ML-DDH presents as a solitary well-defined medallion-shaped erythematous and atrophic patch located on the upper trunk or neck that remains stable over time [13–16]. ML-DDH is histologically characterized by a spindle cell proliferation in the dermis and subcutaneous fat [14–17]. Furthermore, immunohistochemical staining for CD34 and factor XIIIa is usually positive and molecular testing for *COL1A1-PDGFB* fusion gene is absent in ML-DDH [14], which distinguishes ML-DDH from atrophic DFSP.

DFSP is a locally aggressive tumor with a high postsurgical recurrence rate. Surgical resection is the standard treatment for DFSP; however, complete resection is sometimes difficult because the minimum resection margin needed to achieve local control remains undefined [1]. Local recurrences rates were 26–60 % when DFSP was excised with undefined or conservative surgical margins [1]. After wide local excision of 2–3 cm, the reported total local recurrence rate was found to be much lower (0–30 %) [1]. When margins of 5 cm were obtained, the recurrence rate was reduced to <5 % [1]. Increasingly wider margins have resulted in lower recurrence rates; however, obtaining generous margins is not always possible, especially when the tumors are present on the face or neck or in children. In addition, as the surgical margins are extended, reconstruction of the defective area is more complex and may leave an important cosmetic defect. DFSP is a radiosensitive disease with excellent local control after conservative surgery in combination with radiation therapy [18]. Radiation can be considered when there is a concern about incomplete surgical resection, positive surgical margins are found after resection and further surgery is impossible, or the lesions are >5 cm [18].

Metastasis is rare occurring in only 2–5 % of cases, and the most common route is hematogenous dissemination to the lungs [1]. Although it is difficult to determine which cases are at risk of metastasis, cases of metastasis generally involve recurrent lesions that have progressed for many years and a fibrosarcomatous component on histology [1]. Local recurrences of DFSP mostly appear within 3 years of surgery, although late recurrences may occur [1]. Therefore, after surgery, patients should be examined every 6 months for the first 3 years and annually thereafter for life [1]. In the present case, we performed a wide local excision (25–30 mm), and neither recurrence nor metastasis has been observed for 5 years and 8 months since the surgery.

Translocation of t(17;22)(q22;q13) is the cytogenetic feature of DFSP. This chromosomal rearrangement leads to fusion of the collagen type I α1 gene (*COL1A1*) on 17q with the platelet-derived growth factor β gene (*PDGFB*) on 22q [1–5]. In all of the cases described to date, using multiplex RT-PCR, the break point of *PDGFB* is constant (exon 2), whereas in *COL1A1*, the break may occur in any of the exons codifying the alpha-helical region (exons 6–49) [1]. To date, 38 different *COL1A1-PDGFB* fusion variants have been shown to be involved in the pathogenesis of DFSP, with exons 25, 32, and 47 being the most frequently involved [3]. However, no correlation was found between the different *COL1A1* break points within the fusion gene and histopathological subtypes of DFSP or the clinical characteristics [3, 4]. In the present case, sequencing of the multiplex RT-PCR amplification product revealed fusion of exon 25 of *COL1A1* to exon 2 of *PDGFB*. To our knowledge, this is the fourth reported case of atrophic DFSP in which the *COL1A1-PDGFB* fusion gene was detected: Exons 25 [2], 31 [5], and 2 [11] of *COL1A1* were previously reported to be involved in the pathogenesis of atrophic DFSP. These findings imply that even in atrophic DFSP, detection of the *COL1A1-PDGFB* fusion gene is a powerful tool for molecular diagnosis to differentiate other benign/malignant neoplasms.

## Conclusion

Here, we presented the case of a patient with congenital atrophic DFSP with a lesion that had been recognized at birth and remained quiescent for a long period of time before developing into a tumor. This atrophic variant of DFSP is believed to represent an early stage of the lesion, and awareness of this condition can aid in early diagnosis and thereby improve the prognosis of DFSP.

## Consent

Written informed consent was obtained from the patient for publication of this Case Report and any accompanying images. A copy of the written consent is available for review by the Editor-in-Chief of this journal.

## Abbreviations

DFSP: Dermatofibrosarcoma protuberans
RT-PCR: Reverse transcription polymerase chain reaction
ML-DDH: Medallion-like dermal dendrocyte hamartoma
*COL1A1*: The collagen type I α1 gene
*PDGFB*: The platelet-derived growth factor β gene
